# Mobility in Blue-Green Spaces Does Not Predict COVID-19 Transmission: A Global Analysis

**DOI:** 10.3390/ijerph182312567

**Published:** 2021-11-29

**Authors:** Zander S. Venter, Adam Sadilek, Charlotte Stanton, David N. Barton, Kristin Aunan, Sourangsu Chowdhury, Aaron Schneider, Stefano Maria Iacus

**Affiliations:** 1 Norwegian Institute for Nature Research—NINA, Sognsveien 68, 0855 Oslo, Norway; David.Barton@nina.no; 2 Google, Mountain View, CA 94043, USA; sadilekadam@google.com (A.S.); chstanton@google.com (C.S.); aaronschneider@google.com (A.S.); 3 CICERO Center for International Climate Research, P.O. Box 1129 Blindern, N318 Oslo, Norway; kristin.aunan@cicero.oslo.no; 4 Department of Atmospheric Chemistry, Max Planck Institute for Chemistry, 55128 Mainz, Germany; S.Chowdhury@mpic.de; 5 European Commission, Joint Research Centre, 21027 Ispra, Italy; stefano.iacus@ec.europa.eu

**Keywords:** non-pharmaceutical interventions, SARS-CoV-2, outdoor, policy, pollution, recreation, UV

## Abstract

Mobility restrictions during the COVID-19 pandemic ostensibly prevented the public from transmitting the disease in public places, but they also hampered outdoor recreation, despite the importance of blue-green spaces (e.g., parks and natural areas) for physical and mental health. We assess whether restrictions on human movement, particularly in blue-green spaces, affected the transmission of COVID-19. Our assessment uses a spatially resolved dataset of COVID-19 case numbers for 848 administrative units across 153 countries during the first year of the pandemic (February 2020 to February 2021). We measure mobility in blue-green spaces with planetary-scale aggregate and anonymized mobility flows derived from mobile phone tracking data. We then use machine learning forecast models and linear mixed-effects models to explore predictors of COVID-19 growth rates. After controlling for a number of environmental factors, we find no evidence that increased visits to blue-green space increase COVID-19 transmission. By contrast, increases in the total mobility and relaxation of other non-pharmaceutical interventions such as containment and closure policies predict greater transmission. Ultraviolet radiation stands out as the strongest environmental mitigant of COVID-19 spread, while temperature, humidity, wind speed, and ambient air pollution have little to no effect. Taken together, our analyses produce little evidence to support public health policies that restrict citizens from outdoor mobility in blue-green spaces, which corroborates experimental studies showing low risk of outdoor COVID-19 transmission. However, we acknowledge and discuss some of the challenges of big data approaches to ecological regression analyses such as this, and outline promising directions and opportunities for future research.

## 1. Introduction

During 2020, governments around the world took measures to prevent the spread of COVID-19, including non-pharmaceutical interventions that enforced social distancing within the population [1]. In some countries (e.g., Italy), stringent interventions such as stay-at-home or shelter-in-place policies restricted human mobility to indoor residential environments, and resulted in significant reductions in visitation to blue-green spaces (non-built-up areas, such as parks, watercourses, and natural areas, often used for recreation) [2,3,4]. In other countries (e.g., Norway), less severe mobility restrictions allowed citizens to be mobile outdoors while maintaining physical distance and taking personal precautions, including wearing masks and washing hands [5,6,7]. Given the negative consequences that social distancing and home confinement can have on mental and physical health outcomes [8,9,10], and the positive influence of time spent in outdoor recreational areas and parks [11,12], it is important to ensure that the benefits of reduced public transmission due to indoor confinement policies outweigh the negative health impacts of reduced outdoor physical exercise and mental recreation.

Most of the early COVID-19 outbreaks were associated with indoor settings [13,14], and reviews of the scientific evidence accumulated during 2020 show that outdoor transmission of COVID-19 is substantially lower compared to indoor transmission (approximately 19 times lower) [15,16]. Despite this evidence, over 30% of countries maintained some level of stay-at-home requirements, even up to two months after countries started easing restrictions [2]. This is possibly because observational and experimental evidence is often restricted in geographical scope, with context-specific findings that are not easily generalizable. To address this, modelling studies have attempted to rank the effectiveness of non-pharmaceutical interventions on COVID-19 spread on a global scale, however, they have not been able to conclude anything specific about the relative importance of outdoor mobility and indoor confinement policies [1]. This is partly because prior studies did not have access to human mobility data, and partly because such studies did not control for the confounding effect of environmental factors, including air pollution, temperature, humidity, wind speed, and ultraviolet (UV) radiation, which have all been implicated in influencing COVID-19 transmission, morbidity, and mortality [17,18,19,20].

Here, we leverage a unique planetary-scale mobility dataset to quantify the association between mobility in blue-green spaces, environmental factors, and COVID-19 growth rates (a proxy for transmission) over 848 administrative units across most of the world’s countries (153). Specifically, we use regularization statistical methods, machine-learned regression trees and linear mixed modelling to evaluate the relative importance of mobility in blue-green spaces and environmental factors on COVID-19 growth rates between February 2020 and February 2021. We find that mobility in blue-green spaces is a weak predictor of COVID-19 growth rates, whereas total mobility, stringency of non-pharmaceutical interventions and ultraviolet radiation are strong predictors. 

## 2. Methods

### 2.1. Data

#### 2.1.1. COVID-19 Data

We extracted COVID-19 daily case numbers from the COVID-19 Data Hub using the ‘COVID19′ R package [21]. The data were collated from several sources, including the Johns Hopkins University Applied Physics Laboratory. The unique offering of the COVID-19 Data Hub is the provision of data at finer spatial grain; specifically, admin 2 level (county in the US), which includes states, regions, and cantons. Daily cases were aggregated to weekly intervals to match the temporal grain of the Google mobility data (see below). The response variable of interest in this study was defined as the growth rate (λ) of weekly COVID-19 cases (C), as:

$$\lambda_{t}^{C}=ln\left(C_{t}\right)-ln\left(C_{t-1}\right)$$

This variable has been used in a number of epidemiological time series analyses of COVID-19 (e.g., refs. [1,17]) and is a proxy for transmission of the virus. The growth rate variable is also advantageous, because it reduces the confounding effect of differing testing and reporting rates between regions [22]. We made no filter on the COVID-19 dataset to exclude administrative units, which had very low case numbers from the start of the pandemic (e.g., New Zealand), because we used statistical measures to account for this effect.

#### 2.1.2. Aggregate Mobility Data

The Google COVID-19 Aggregated Mobility Research Dataset contains anonymized mobility flows aggregated over users who have turned on the Location History setting, which is off by default. This is similar to the data used to show how busy certain types of places are in Google Maps—helping detect when a local business tends to be the most crowded. The dataset aggregates the flows of people from region to region defined by S2 cells of size approximately 5 km^2^ (https://github.com/google/s2geometry, accessed on 15 October 2021). To provide strong privacy guarantees, all trips were anonymized and aggregated using a differentially private mechanism [23] to aggregate flows over time (see https://policies.google.com/technologies/anonymization, accessed on 15 October 2021). This research is done on the resulting heavily aggregated and differentially private data. A description of the production of the Google mobility dataset is provided in the Supplementary Materials.

We calculated total mobility (TM) as the sum of in- and out-flows (which include within-cell trips defined as a self-loop in the mobility graph) for all S2 grid cells, falling within each administrative unit (x).

$$TM_{x}=\overset{n}{\underset{i=1}{\sum }}Inflow_{i}+Outflow_{i}$$

Due to privacy requirements, it is not possible to distinguish flow vectors that originate and terminate indoors (grey space) or outdoors (blue-green spaces). Therefore, to quantify blue-green space mobility, total mobility flows were weighted by the proportion of blue-green space (bgf) within their respective S2 grid cells. Here, we assume that mobility flows in areas with greater proportions of blue-green space, as opposed to mobility in built-up grey spaces, are more likely to represent outdoor activity in parks, beaches, marinas, gardens, and other private or public spaces. Blue-green space was defined as any non-artificial land cover, where artificial land was extracted from the Global Urban Footprint global dataset [24] at 12 m spatial resolution (Figure S1). This includes all open spaces that are covered by vegetation, bare ground, or water (water body surface area further than 500 m off the shoreline was excluded). Cropland was included in our definition of blue-green space, due to the importance of agricultural landscapes for recreational activities [5]. Nevertheless, we performed a separate analysis with cropland, defined at 10 m resolution by the ESA WorldCover dataset [25], excluded from blue-green spaces, and found no change in the significance of blue-green mobility as a predictor of COVID-19 growth rates (Figure S2). Weekly blue-green space mobility (BGM) for administrative unit x was defined by aggregating S2 grid cells as follows:

$$BGM_{x}=\frac{\sum_{i=1}^{n}TM_{i}\times bgf_{i}}{\sum_{i=1}^{n}TM_{i}\;}$$

Total and blue-green mobility for administrative units for each week in the time series November 2019 to February 2021 were calculated (spatial and temporal scope defined by the COVID-19 dataset; Figure S3). 

#### 2.1.3. Covariate Data

Data on a range of covariates that are expected to influence or mediate the interaction between mobility and COVID-19 transmission were collected on a global scale. The spatial grain of covariate data differed to that of the mobility data above, however all data were aggregated up to a common spatial unit (administrative level 2) defined by the COVID-19 data.

Gridded hourly temperature, relative humidity, wind speed and ultraviolet (UV) radiation data were collected from the European Centre for Medium-Range Weather Forecasts Reanalysis 5th (ERA5) product [26] at 1 arc degree resolution. The suite of climatic covariates was chosen because they are the most commonly cited as factors influencing COVID-19 [17,19]. Data on ambient particulate matter with a diameter less than 2.5 μm (PM2.5) were collected from the Copernicus Atmosphere Monitoring Service [27] at 0.4 arc degrees resolution. PM2.5 was chosen because it is the main pollutant implicated in enhancing the spread and lethality of SARS-CoV-2 [28]. We aggregated the climate and pollution data to each administrative unit by calculating population-weighted means. Population data were obtained for 2020 from the Gridded Population of the World v4 dataset [29]. 

In addition to environmental covariates, we also collected data on non-pharmaceutical policy interventions collated by the Oxford Covid-19 Government Response Tracker, which include measures of government response, containment, stringency, risk of openness [2]. The stringency of daily policy interventions have been collated into a stringency index, which reflects a range of containment, closure and health system policies. In an attempt to control for the spatio-temporal variability in COVID-19 testing rates, we collected time series data on the total number of tests per thousand people from ref. [22].

We collected ancillary demographic and health variables that are important determinants of COVID-19 severity and mortality. These included the baseline death rate, due to communicable diseases provided by the Institute for Health Metrics and Evaluation [30]; the percentage of population above 65 years defined by the Gridded Population of the World dataset [29].

#### 2.1.4. Statistical Techniques

All explanatory data were aggregated up to the spatial (administrative level 2) and temporal (weekly) grain of the COVID-19 data, and limited to the period February 2020 through February 2021. The final dataset consists of 848 administrative units across 153 countries (Figure S3). We statistically estimate the effect of blue-green space mobility on COVID-19 growth rates using both a predictive and explanatory modelling framework. The former relies on machine learning models that forecast COVID-19 growth rates and simultaneously rank the relative importance of predictor variables, while the latter relies on linear mixed-effect modelling, where effect sizes of explanatory variables are estimated while controlling for the effect of others.

In attempting to test the effect of blue-green space mobility on COVID-19 growth rates, we needed to account for several confounding factors which may invalidate our statistical models. Firstly, the location-specific social, economic or environmental factors, which likely contribute to COVID-19 transmission, may also correlate with average mobility levels. Therefore, we code for site-specific fixed-effects which flexibly control for spatial variation in confounding factors and data quality across geospatial administrative units. Secondly, COVID-19 growth rate time series may be temporally and spatially correlated, and thereby violate the assumption of non-independence of residuals (for linear models). Therefore, we explicitly code for this autocorrelation structure in our models. Thirdly, the effect of any environmental or mobility variable on COVID-19 growth rates will appear with some delay, due to both the incubation period and the time required to diagnose the disease. Empirical evidence shows that there is a lag of approximately one week between infection and symptom onset [31], and another week delay until case confirmation [32], although these periods will vary between regions and over time. Therefore, we established temporally distributed lag regression models with one, two and three week lagged-effects. We average across the lagged-effects to calculate a cumulative effect as our main statistic of interest. Finally, COVID-19 testing and reporting rates vary substantially over space and time [22]. Although our location-specific fixed effects control for this somewhat, we also include time series of testing rates per country to account for this variability. 

#### 2.1.5. Combination of Elastic Net and Random Forest Modelling

The predictive modelling workflow was conducted using the ‘caret’ machine learning package in R [33]. We first estimated an elastic net model using all environmental, policy and mobility predictors. Elastic net is a regularized regression method that linearly combines the lasso and ridge penalization methods [34] to simplify the set of model predictors. Once an optimal subset of predictors was established, we estimated a random forest regression model [35] to rank the importance of predictor variables in forecasting COVID-19 growth rates. Random forest was chosen for this step because, unlike elastic net, it accounts for non-linear relationships between predictor and response variables. We control for spatial and temporal autocorrelation by adopting a k-fold cross-validation, using the administrative unit and week to define the spatial and temporal validation folds, respectively. We iteratively built models on two-month blocks of data over the study period to quantify the variation in predictor variable importance over time.

#### 2.1.6. Linear Mixed-Effects Modelling

The explanatory modelling framework was conducted using the ‘lme’ package in R [36]. Linear mixed-effects models allowed us to estimate the direction and magnitude of predictor effects on COVID-19 growth rates after controlling for random effects. It also serves as a form of sensitivity analysis, because we expect the magnitude of variable effects to correspond to the variable importance rankings in the machine learning models described above. Total and blue-green space mobility, the climatic variables, policy stringency index, testing rate, percentage of elderly, and baseline communicable disease mortality rates were included as fixed effects. Week and administrative unit were assigned as random effects. We defined a spatial and temporal autocorrelation structure with administrative unit ID and week. To explicitly account for the spatial variation in reporting rates, which is correlated with national GDP [22], we stratified the linear modelling to include administrative units from low-, mid- and high-income countries. To account for the different epidemiological dynamics operating during the first and proceeding waves of confirmed cases, we further stratified the modelling into pre- and post- June 2020.

## 3. Results

To estimate the relative effects of blue-green space mobility, environmental and policy variables on COVID-19 growth rates, we used predictive and explanatory modelling frameworks. With the predictive modelling framework, we calibrated two types of machine learning models to assess the relative importance of predictor variables, using a sliding time-window to train the models and move one step forward each week. An elastic net model [34] was fitted first to estimate an optimal subset of predictors, through a balanced regularization method that leverages both lasso and ridge regression. Subsequently, we fitted a random forest regression model [35], which accounts for non-linearity effects of predictor variables, to calculate the relative importance of each predictor. Relative importance is calculated by iteratively removing each variable from the model, and calculating the relative drop in predictive accuracy.

While elastic net and random forest machine learning models provide insight into the relative importance of predictor variables, they are not flexible enough to comprehensively control for confounding factors and location-specific “fixed effects”. We therefore used an explanatory modelling framework, and specifically, linear mixed-effects models, to estimate the magnitude and direction of predictor effects on COVID-19 growth rates. Our mixed-effects models flexibly controlled for (1) location-specific confounders such as socio-economic, environmental, climatic and data quality characteristics that vary across geospatial units; (2) spatial and temporal autocorrelation; (3) and the lagged effects on COVID-19 growth rates. We stratified the models over space and time to aid interpretability of their outputs, and to account for different levels of case reporting and pandemic response. Firstly, we used country GDP to stratify administrative units spatially, given that a range of pandemic responses vary substantially with GDP [22], not least of which is testing and reporting rate. Secondly, government and public responses to the pandemic varied substantially over time, and therefore we attempted to separate the period, including the first waves of COVID-19 cases (pre-June 2020) from the latter period, including subsequent waves.

### 3.1. Human Mobility and COVID-19 Transmission

**Figure 1:**
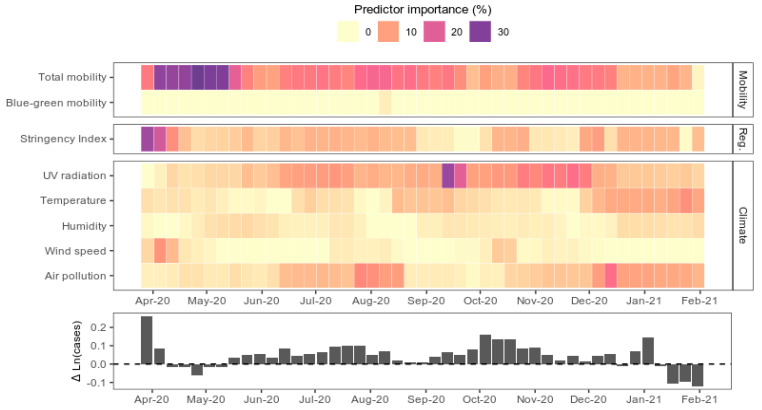
Relative importance of predictor variables in machine learning model forecasts of COVID-19 growth rates. The colour saturation of weekly blocks reflects the relative importance of predictor variables in forecast models trained on the preceding two months of data. Importance is defined as the decrease in model prediction accuracy when the variable in question is omitted from the model. An elastic net model was fitted to select the best predictors for a given time window. The selected variables were then used to build a random forest model, to assess the relative importance of each variable while accounting for non-linear effects. The response variable, COVID-19 growth rate aggregated at the global level, is plotted in the lower panel for reference.

Total mobility was the most important variable for forecasting COVID-19 transmission averaged over the course of the first year of the pandemic (Figure 1). The variable importance score, defined as the reduction in model prediction accuracy when the variable is omitted from the model, for total mobility, was 15 ± 6.8% (±standard deviation). It was particularly important during the first waves of COVID-19 cases (21% importance in pre-June period), but was less important later on (dropping to 12% post-June). In contrast, the proportion of mobility in blue-green spaces was substantially less important (0.5 ± 0.3%) for predicting transmission throughout the year.

**Figure 2:**
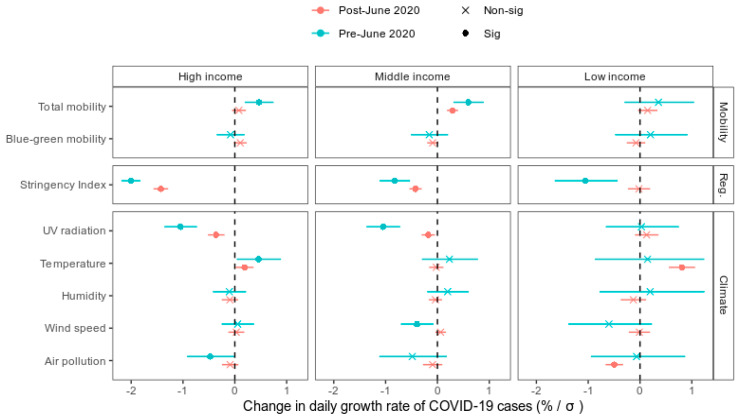
Empirical estimates of the association between COVID-19 growth rates and mobility, restrictions and environmental conditions. The cumulative effect of each predictor variable is derived from mixed-effects linear regression models built for high-, middle- and low-income countries, pre- and post-June 2020. Points and lines represent the model estimates and 95% confidence intervals. Non-significant (“non-sig”) estimates are marked with an “x” whereas significant (“sig”) estimates are marked with a solid point. Estimates are expressed as percentage changes in daily COVID-19 cases per standard deviation (δ) increase in the predictor variable.

The relative importance of total and blue-green space mobility is reflected in the coefficient magnitudes from the linear mixed-models (Figure 2) that controlled for confounding factors. The explanatory modelling framework revealed that every one standard deviation (SD) increase in total mobility resulted in a 0.3% (0.05 to 0.6%; 95% confidence interval) increase in COVID-19 growth rate. This effect was more apparent in middle income compared to high- and low-income countries, and particularly during the first waves of the pandemic, pre-June 2020. In contrast, the proportion of mobility in blue-green space did not have a statistically significant effect (*p* < 0.05) on COVID-19 growth rates across income and temporal strata (Figure 2). We also tested the association in a model which excluded agricultural land in the definition of blue-green space, and found no change in the significance of blue-green mobility as a predictor of COVID-19 growth rates (Figure S2).

### 3.2. Policy Interventions Facilitate the Mobility Effect

The stringency of non-pharmaceutical policy interventions, defined by the “stringency index”, was also a strong predictor of COVID-19 transmission (6.6 ± 4.8%), particularly pre-June 2020, when it was ranked as the most important predictor (Figure 1). The stringency index encapsulates a range of containment, closure and health system policies collated by the Oxford Covid-19 Government Response Tracker [2]. For every standard deviation increase in the stringency index, COVID-19 growth rates decreased by 0.96% (0.7 to 1.2%) (Figure 2). This effect was diminished in lower income countries, particularly post-June. The stringency index is linearly associated with declines in total mobility (Figure S4), which suggests that policy interventions mitigate COVID-19 growth rates via their effect on human mobility.

### 3.3. Environmental Covariates of COVID-19 Transmission

UV radiation was the second most important predictor overall, and the most important out of the set of climatic predictors (Figure 1) in the COVID-19 growth forecast models. Removing UV radiation reduced the model accuracies by 8.7 ± 4.9%. It was particularly important during Oct–Nov when growth rates were peaking globally. Linear models revealed that the direction of the UV effect was negative; for every 1 standard deviation increase in UV radiation, there was a 0.4% (0.1 to 0.7%) decrease in the COVID-19 growth rate (Figure 2). However, in low-income countries specifically, the negative effect of UV was not significant. 

Compared to UV radiation, the other environmental variables including wind speed, temperature, humidity and air pollution were weak predictors of COVID-19 growth rates (Figure 1). Although temperature and ambient air pollution (fine particulate matter) were more important predictors than UV at certain times of year (Figure 1), the overall significance and direction of their effect was less clear (Figure 2). Temperature increases were associated with elevated COVID-19 growth rates in high-income countries, and post-June in low-income countries. Air pollution was negatively associated with COVID-19 growth rates pre-June in high-income countries, and post-June in low-income countries.

## 4. Discussion

Our global-scale modelling study provides supporting evidence for local-scale experimental and observational studies [15,16], which show that higher mobility in outdoor blue-green spaces is not associated with enhanced risk of COVID-19 transmission. It also corroborates other modelling studies showing park closures [1] and declines in outdoor park visitation [37] are not significant predictors of COVID-19 spread. However, we also identify important challenges and limitations to global ecological regression analyses such as ours, which are discussed further down in this section.

Our statistical models show that the lack of an outdoor mobility effect on COVID-19 growth rates persists, even after accounting for potential confounders, including meteorological factors known to influence epidemic dynamics. In contrast, reductions in total population mobility facilitated by non-pharmaceutical interventions were a strong predictor of negative COVID-19 growth rates in our analysis, confirming observational studies in China [38] and the USA [39]. However, in low-income countries, growth rates were less responsive to population mobility or confinement policies compared to middle- to high-income countries (Figure 2). This may be because poverty generally reduces compliance with COVID-19 shelter-in-place policies [40], or because the underreporting of COVID-19 cases in low-income countries [41] adds noise to our dataset which was not adequately accounted for by the variables in our statistical models. Across all GDP brackets, we found that growth rates were more responsive to mobility and policy interventions during the initial pandemic outbreak (pre-June 2020) compared to after (Figure 1 and Figure 2). This may be due to pandemic policy fatigue [42], in which adherence to governments’ protective-behaviour policies against COVID-19 diminishes with time.

The primary implication of our findings, which contributes to existing knowledge on mobility restrictions, is that restricting citizens from recreating outdoors in blue-green spaces may not be necessary to curb the spread of COVID-19. Indeed, the most effective government interventions to date, which include restricting long-distance mobility (i.e., border restrictions), social gatherings, and curfews [1], do not necessitate restricting outdoor mobility. Less disruptive and costly interventions, such as public awareness and education campaigns about personal protection measures, can be as effective as more intrusive ones [43]. Furthermore, the cost of confining citizens indoors is a substantial public mental and physical health burden. A review of the recent epidemiological literature shows that lockdown measures during the pandemic are significantly associated with increased stress, anxiety, depressive symptoms, social isolation, and psychological distress [10]. In contrast, having access to blue-green space during the pandemic has been associated with enhanced mental and emotional wellbeing and reduced stress [11]. By restricting outdoor mobility, governments may be inadvertently exacerbating socio-economic inequalities [44], particularly in countries where poorer citizens do not have access to private green space like gardens (e.g., South Africa [45]).

Despite the value of mobility in blue-green spaces, we emphasize that routine personal protection measures should be maintained during outdoor activity, given that COVID-19 transmission remains possible outdoors [13]. Specifically, factors including a lack of personal protective equipment (e.g., face masks), duration, proximity and frequency of personal contact, and occasional indoor gathering during a largely outdoor experience have been associated with outdoor reports of infection [16]. Therefore, initiatives to keep parks and recreational areas open should be tempered with public health awareness campaigns that promote social distancing and personal protection [12]. Furthermore, outdoor activity enhances exposure to environmental factors which may mitigate or facilitate viral transmission. In line with other global modelling studies [17], we found that UV radiation was the strongest environmental factor predicting declines in COVID-19 growth rates (Figure 2). In contrast to national- and regional-scale modelling studies, we find no evidence to support the hypothesis that fine particulate matter air pollution facilitates viral transmission [28]. Therefore, recreation outdoors during the pandemic may be safer on sunny days with greater UV radiation levels.

The results in our study should be interpreted in light of a few important limitations that are common to “big data” approaches to epidemiological modelling. The Google mobility data are limited to smartphone users who have opted in to Google’s Location History feature, which is off by default. As a result, these data may not be representative of the population as a whole, and representativeness may further vary by location. Comparisons across, rather than within, locations are only descriptive, since these regions can differ in substantial ways significant for transmission, such as income levels. However, we expect representativeness of the mobility data to be relatively homogenous within low-, middle- and high-income state groupings (e.g., smartphone purchasing rates and cellular network coverage are generally lower in low-income countries), and therefore, our stratification of the modelling (see Figure 2) accounts for this. In addition, the Google mobility data is more robust over time for a given location than between different locations. Our modelling framework also relies on the temporal variation in mobility to explain COVID-19 growth rates, and this component of the model is therefore robust against issues of spatial representativeness.

Another limitation to our analysis, which is shared with ecological regression approaches to epidemiological modelling, is that strictly causal inference is not possible. This is primarily due to the complexities of virus transmission, and the fact that we are inevitably missing other confounding variables that could limit the interpretation of the results. For instance, the influence of blue-green space characteristics (e.g., size, quality, amenities) was not captured in our model, due to privacy constraints in the mobility data which do not allow identifying exact origin and destination locations for trips within a 5 km^2^ mobility grid cell. This may be significant, because visitation to small pocket parks, which were closed in many cities during the pandemic, were not captured in our data. Therefore, we acknowledge that our data cannot be used to make conclusions on the influence of walking distance blue-green spaces on COVID-19 transmission. The privacy settings on mobility flows also mean that we cannot fully distinguish between changes in behaviour for shopping for essentials, shopping for non-essentials, or for blue-green space use. Our results rest on the assumption that mobility flows in areas with greater proportions of blue-green space are more likely to represent outdoor activity trips that either originate, end, or follow paths in blue-green spaces. Our results also assume that the area of blue-green space within a mobility grid cell as a proxy for exposure; however, recreational use may be disproportionate to available blue-green space area. For example, cropland is largely private land inaccessible to the public, apart from roads that pass through it, and its surface area is likely not proportional to its use. We did test this by excluding cropland from the definition of blue-green space, and found no change in the overall result (Figure S2). However, this may be an issue common to other land-use classes, such as protected watercourses or private gardens.

Ecological regression approaches like ours also rely on aggregated data (up to administrative spatial units) which cannot be used to infer anything about individual-level associations between blue-green space and COVID-19 transmission, because doing so would lead to the “ecological fallacy” [46]. The same is true for the predictor variable blue-green space mobility; aggregation to a coarser spatial unit assumes that everyone in that area experiences the same exposure to blue-green space.

Despite the limitations discussed above, our modelling approach remains useful as a form of hypothesis generation and corroboration of evidence from experimental studies. The challenges of ecological regression approaches also provide scope for future research. In the context of our study, these may include performing experimental cohort studies that monitor individual viral load after mobility, through blue-green spaces of varying size and design. Conducting experimental work at the individual level allows for adequate controls of confounding variables. Experimental work will also allow for testing our assumption that mobility flows weighted by blue-green space do, in fact, capture the spatial and temporal variations in actual human exposure to blue-green space.

In conclusion, we offer unique evidence, at the global scale, that supports observational studies, showing that mobility in outdoor blue-green spaces is associated with no significant additional risk of COVID-19 transmission. Results are based on a spatially resolved dataset of 848 administrative units across 153 countries during the first year of the pandemic. Nevertheless, we encounter limitations to the “big data” regression approach which highlight the importance of conducting experimental intervention studies and collecting individual-level mobility and health response data, albeit difficult during a global pandemic. Severe confinement policies like shelter-in-place, that prevent outdoor mobility, may not be justified given our findings, the evidence from experimental studies, and the collateral damage to public mental health.

## Data Availability

The Google COVID-19 Aggregated Mobility Research Dataset used for this study is available with permission from Google LLC. The COVID-19 case data can be downloaded at https://covid19datahub.io/ (accessed on 15 October 2021). The climate data can be found at https://cds.climate.copernicus.eu/cdsapp (accessed on 15 October 2021). Air pollution data can be accessed via https://atmosphere.copernicus.eu/ (accessed on 15 October 2021). Policy response data can be obtained from https://github.com/OxCGRT/covid-policy-tracker (accessed on 15 October 2021). Baseline death rates due to communicable disease can be found at http://ghdx.healthdata.org/ (accessed on 15 October 2021). Gridded population data can be accessed via https://sedac.ciesin.columbia.edu/data/collection/gpw-v4 (accessed on 15 October 2021). Country GDP data are available at https://data.worldbank.org/ (accessed on 15 October 2021).

## Supplementary Materials

The following are available online at https://www.mdpi.com/article/10.3390/ijerph182312567/s1, Supplementary Methods, Figure S1: Spatial grain of data used to define blue-green space, Figure S2: Effect of including cropland in the definition of blue-green space, Figure S3: Spatial and temporal stratification and extent of the data used in the present study, Figure S4: Association between total mobility changes and government stringency index.
