# Supplementary material for: Mobility in Blue-Green Spaces Does Not Predict COVID-19 Transmission: A Global Analysis
Source: Int J Environ Res Public Health. 2021 Nov 29;18(23):12567. doi: 10.3390/ijerph182312567 (PMC8656877; doi:10.3390/ijerph182312567)
Supplement: Supplementary file 1 [file ijerph-18-12567-s001.zip › ijerph-1491330-supplementary.pdf]

Supplementary methods

This section describes the pre-processing of the Google Aggregated Mobility Research Dataset in more detail. To create this dataset, machine learning is applied to logs of GPS trace data to automatically segment it into semantic trips [1]. To create this dataset, a series of rule-based clustering and routing models are applied to logs data to automatically segment it into semantic trips. The details of the semantic segmentation of unique trips are explained by Kirmse et al. [2], but we summarize it in brief here. First, the raw GPS point logs are filtered for erroneous points including those with localities outside the boundaries of international time zones over land, implausible timestamps, and where velocities between consecutive points indicate "jitter" caused by device malfunctions and connectivity issues. The filtered GPS points are then run through a series of clustering algorithms including lead-based clustering, mean-shift clustering, and adaptive radius clustering, to distinguish dwell points from commute points. The commute points are then used to define trips between origin and destination point clusters. Commute points are snapped to road or trail paths using the Google routing engine which is also used to compute driving directions on Google Maps. Commute trips both within and between S2 grid cells are used to derive the weekly aggregated mobility flows used in our analysis.

To provide strong privacy guarantees, all trips were anonymized and aggregated using a differentially private mechanism[3] to aggregate flows over time (see https://policies.google.com/technologies/anonymization). Therefore, our research is done on the resulting heavily aggregated and differentially private data. No individual user data were ever manually inspected, only heavily aggregated flows of large populations were handled.

All anonymized trips are processed in aggregate to extract their origin and destination location and time. For example, if users traveled from location a to location b within time interval t, the corresponding cell (a,b,t) in the tensor would be $n \mp err$, where err is Laplacian noise. The automated Laplace mechanism adds random noise drawn from a zero mean Laplace distribution and yields (ϵ, δ)-differential privacy guarantee of $\epsilon = 0.66$ and $\delta = 2.1 \times 10{-}29$ per metric. Specifically, for each week (W) and each location pair (A,B), we compute the number of unique users who took a trip from location A to location B during week W. To each of these metrics, we add Laplace noise from a zero-mean distribution of scale 1/0.66. We then remove all metrics for which the noisy number of users is lower than 100, following the process described in ref.[3], and publish the rest. This yields that each metric we publish satisfies (ε,δ)-differential privacy with values defined above. The parameter ϵ controls the noise intensity in terms of its variance, while δ represents the deviation from pure ϵ-privacy. The closer they are to zero, the stronger the privacy guarantees.

1. Bassolas, A.; Barbosa-Filho, H.; Dickinson, B.; Dotiwalla, X.; Eastham, P.; Gallotti, R.; Ghoshal, G.; Gipson, B.; Hazarie, S.A.; Kautz, H.; et al. Hierarchical Organization of Urban Mobility and Its Connection with City Livability. *Nat Commun* **2019**, *10*, 4817, doi:10.1038/s41467-019-12809-y.
2. Kirmse, A.; Udeshi, T.; Bellver, P.; Shuma, J. Extracting Patterns from Location History.; 2011; pp. 397–400.
3. Wilson, R.J.; Zhang, C.Y.; Lam, W.; Desfontaines, D.; Simmons-Marengo, D.; Gipson, B. Differentially Private Sql with Bounded User Contribution. *Proceedings on privacy enhancing technologies* **2020**, *2020*, 230–250.

Supplementary figures

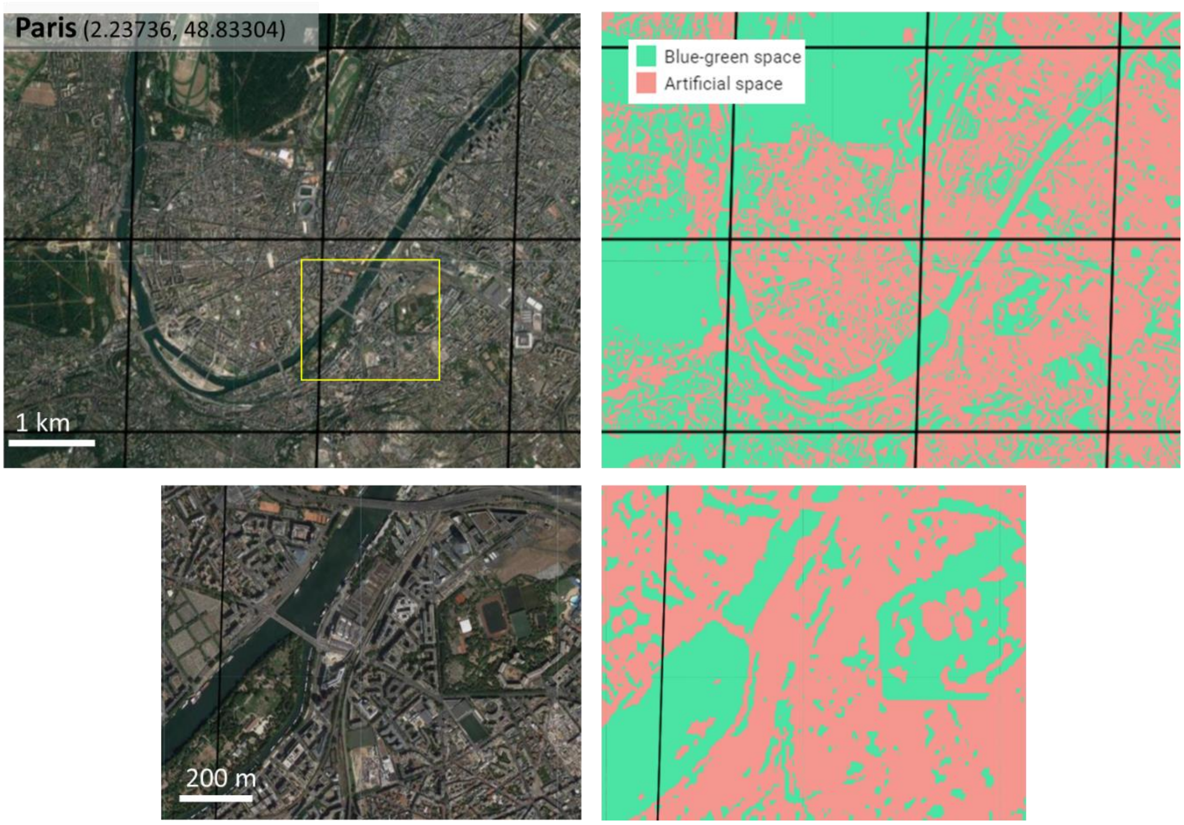

Figure S1. Spatial grain of data used to define blue-green space. Satellite imagery of an urban landscape in Paris, France shown in top left with a zoomed perspective of the area outlined in yellow shown bottom left. The 5km^2 grid used to aggregate mobility data is shown in black.

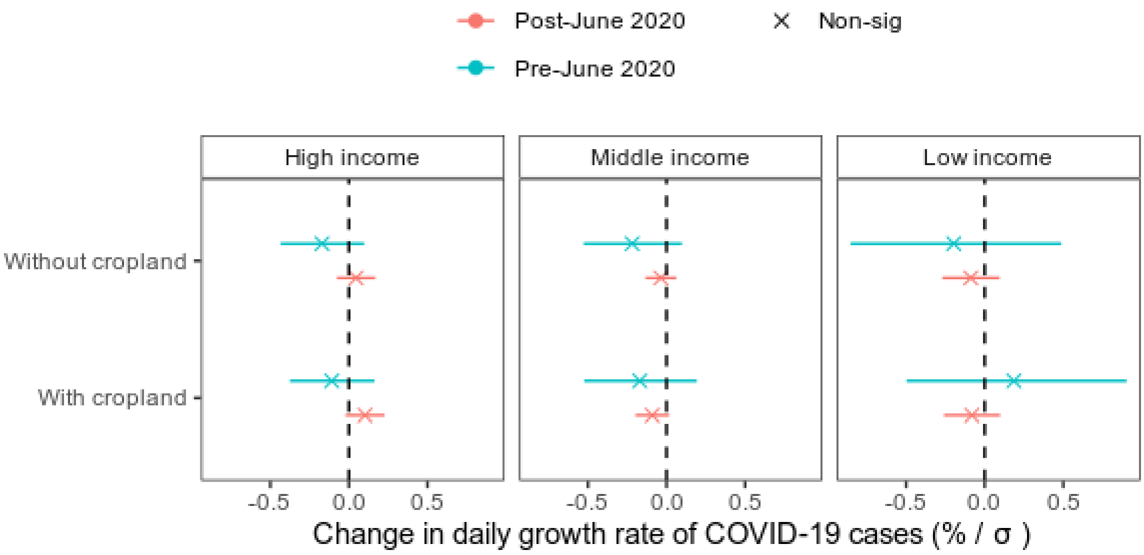

Figure S2. Effect of including cropland in the definition of blue-green space. Empirical estimates of the association between COVID-19 growth rates and blue-green mobility, defined by blue-green space with and without cropland. The cumulative effect of each predictor variable is derived from mixed-effects linear regression models built for high, middle and low income countries pre- and post-June 2020. Points and lines represent the model estimates and 95% confidence intervals. Non-significant estimates are marked with an "x". Estimates are expressed as percentage changes in daily COVID-19 cases per standard deviation (δ) increase in the predictor variable.

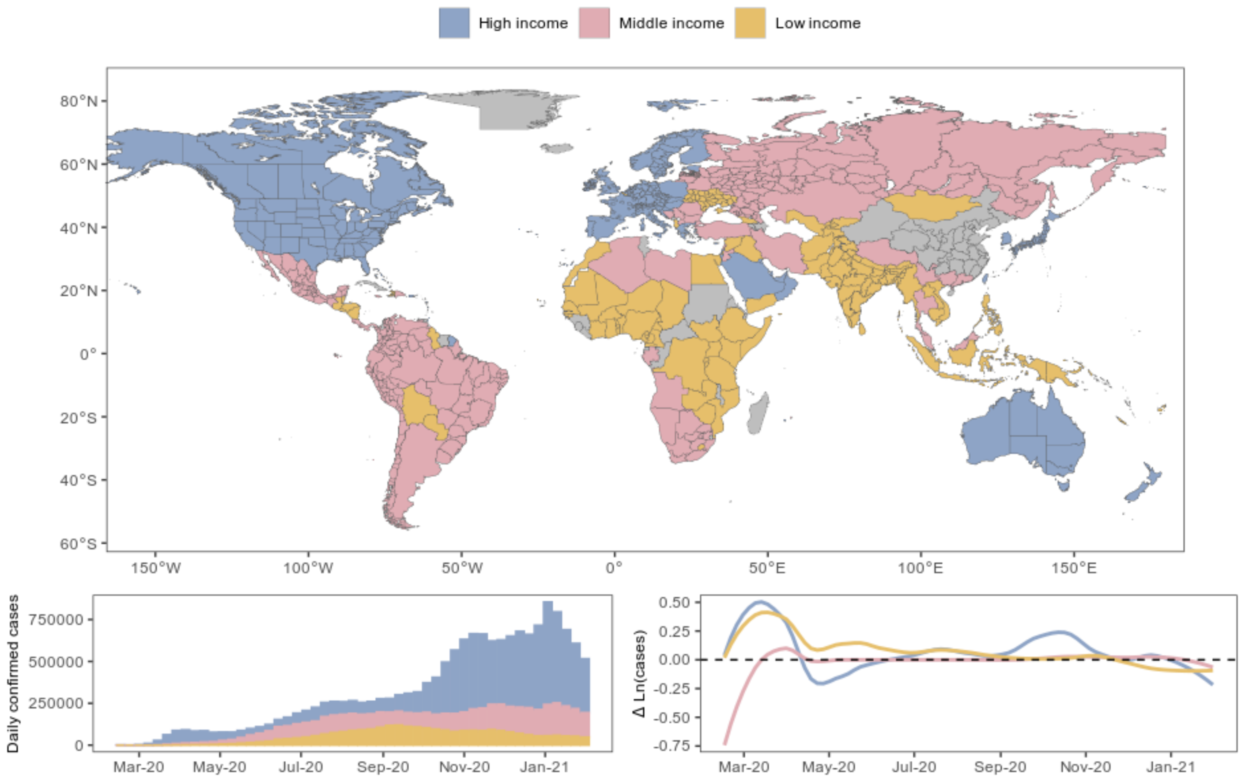

Figure S3. Spatial and temporal stratification and extent of the data used in the present study. The global map shows administrative geographical units used for data aggregation and the distribution of low-, middle- and high-income states. Areas in grey did not contain sufficient COVID-19 or mobility data to be included in the analysis. The weekly time series graphs below show the average daily cases and case growth rates per week during Feb 2020 to Feb 2021.

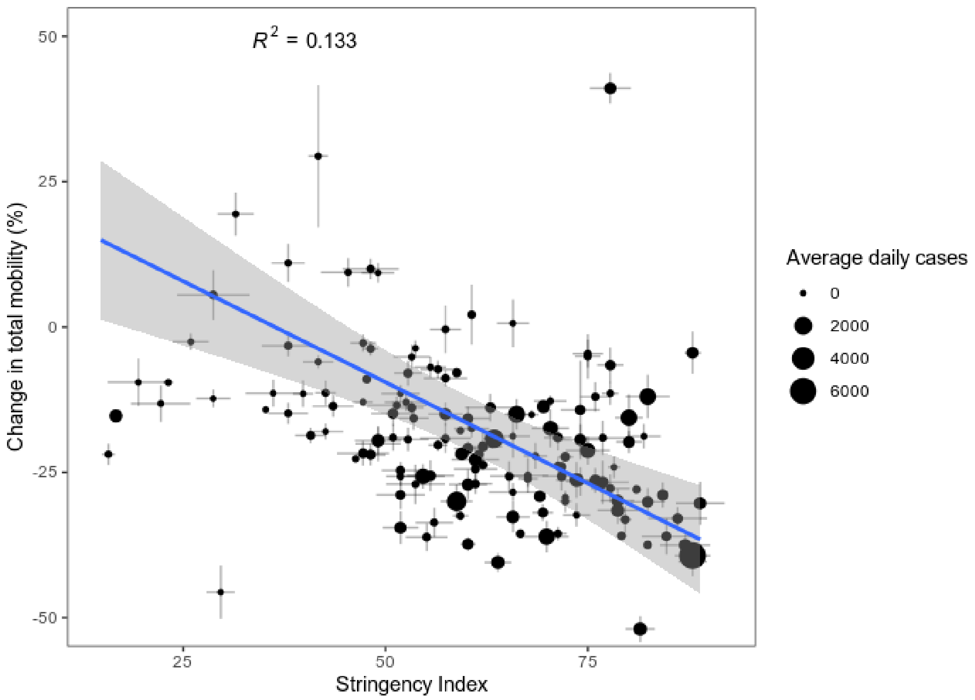

Figure S4. Association between total mobility changes and government stringency index. Each data point represents a unique geographical unit. The size of the point represents the average daily COVID-19 case numbers. Vertical and horizontal whiskers represent the standard error across weekly time series of mobility changes and stringency index. Changes in mobility are expressed in terms of relative differences from a Nov-19 to Feb-20 median baseline value. Stringency index is derived from a combination of containment, closure and health system policies. A linear regression line is fitted in blue with 95% confidence intervals as ribbons. The R^2 value of the linear fit is displayed.
